# New perspectives on biology, disease progression, and therapy response of head and neck cancer gained from single cell RNA sequencing and spatial transcriptomics

**DOI:** 10.32604/or.2023.044774

**Published:** 2023-11-15

**Authors:** GERWIN HELLER, THORSTEN FUEREDER, ALEXANDER MICHAEL GRANDITS, ROTRAUD WIESER

**Affiliations:** 1 Division of Oncology, Department of Medicine I, Medical University of Vienna, Vienna, 1090, Austria; 2 Ludwig Boltzmann Institute for Hematology and Oncology, Medical University of Vienna, Vienna, 1090, Austria

**Keywords:** Head and neck squamous cell carcinoma, Tumor microenvironment, Immunotherapy, Gene expression, Omics

## Abstract

Head and neck squamous cell carcinoma (HNSCC) is one of the most frequent cancers worldwide. The main risk factors are consumption of tobacco products and alcohol, as well as infection with human papilloma virus. Approved therapeutic options comprise surgery, radiation, chemotherapy, targeted therapy through epidermal growth factor receptor inhibition, and immunotherapy, but outcome has remained unsatisfactory due to recurrence rates of ~50% and the frequent occurrence of second primaries. The availability of the human genome sequence at the beginning of the millennium heralded the omics era, in which rapid technological progress has advanced our knowledge of the molecular biology of malignant diseases, including HNSCC, at an unprecedented pace. Initially, microarray-based methods, followed by approaches based on next-generation sequencing, were applied to study the genetics, epigenetics, and gene expression patterns of bulk tumors. More recently, the advent of single-cell RNA sequencing (scRNAseq) and spatial transcriptomics methods has facilitated the investigation of the heterogeneity between and within different cell populations in the tumor microenvironment (e.g., cancer cells, fibroblasts, immune cells, endothelial cells), led to the discovery of novel cell types, and advanced the discovery of cell-cell communication within tumors. This review provides an overview of scRNAseq, spatial transcriptomics, and the associated bioinformatics methods, and summarizes how their application has promoted our understanding of the emergence, composition, progression, and therapy responsiveness of, and intercellular signaling within, HNSCC.

## Introduction

Head and neck squamous cell carcinoma (HNSCC) represents a major health burden worldwide, affecting predominantly males (ratio 4:1) [1]. It is the seventh most common cancer, leading to approximately 325,000 annual deaths globally [2]. In high-income countries, the most common HNSCC subsites comprise the oral cavity, oropharynx, hypopharynx, and larynx. Well-recognised risk factors include consumption of tobacco products and/or alcohol, betel nut chewing, and human papilloma virus (HPV) infection [3,4]. Oral dysbiosis, *Candida albicans* infection, and bacterial genera such as *Fusobacterium*, *Capnocytophaga*, *Prevotella*, *Treponema*, and *Peptostreptococcus* may also contribute to the development of HNSCC [5,6]. HPV^+^ disease, triggered by infection with high-risk variants such as HPV-16 or HPV-18, is mainly confined to the oropharynx, and responsible for the rise of HNSCC incidence in high-income countries in recent decades [2,7]. HPV^+^ oropharyngeal SCC (OPSCC) has a more favorable prognosis compared to HPV^−^ OPSCC irrespective of the treatment modality applied [7,8].

A multidisciplinary approach involving head and neck surgeons, radiation oncologists, and medical oncologists is essential for the optimal management of patients with HNSCC. Surgery, radiotherapy, and systemic therapy are regarded as the standard of care treatment options depending on the subsite and disease stage [9]. However, despite curative intent multi-modality treatment of locally advanced (stage III/IV) HNSCC and the development of novel compounds, recurrence rates remain high (approximately 40%–50%) and second primaries are observed at a constant rate of 2%–3% per year [10,11]. The only oncoprotein-targeting drug approved for HNSCC is the epidermal growth factor receptor (EGFR) antibody cetuximab, which was certified based on a phase III trial more than a decade ago [12]. More recently, the role of cetuximab plus radiotherapy has been challenged, since it was inferior to chemoradiation (CRT) with cisplatin in HPV^+^ OPSCC patients [13,14].

The advent of immune checkpoint inhibitors (ICI) such as pembrolizumab, nivolumab, and avelumab, which target the interactions between programmed cell death protein 1 (PD-1) and programmed death-ligand 1 (PD-L1) and PD-L2, opened new possibilities for combination therapies that might improve the outcomes of patients with locally advanced HNSCC. However, the primary endpoints of several phase III studies comparing standard-of-care CRT to CRT plus ICI were not met [15–17]. The difference in 24-month event-free survival between CRT plus pembrolizumab *vs*. CRT alone (63.2% *vs*. 56.2%) in the KEYNOTE-412 study did not reach significance, but subgroup analysis suggested benefits in patients with PD-L1 combined positive score ≥1 and ≥20% [16]. Promising results were also reported for neoadjuvant and peri-operative ICI, but so far only from smaller trials [18,19].

Among patients suffering from recurrent/metastatic disease not amenable to local salvage therapy, for whom ICI is approved, outcome remained poor with a median overall survival (OS) between 12 and 14 months in KEYNOTE-048, the trial that showed superiority over the former standard of care containing a platinum drug, 5-fluorouracil, and cetuximab (EXTREME regimen) [20]. Generally, only a minority of patients at this stage responded to pembrolizumab and achieved long-term survival. The overall response rate in the total patient population was approximately 17% and 36% for pembrolizumab or pembrolizumab plus chemotherapy, respectively, and 5-year OS was only around 20% for the latter group [20,21]. In summary, primary and secondary resistance to ICI is common, resulting in disease progression.

**Figure 1:**
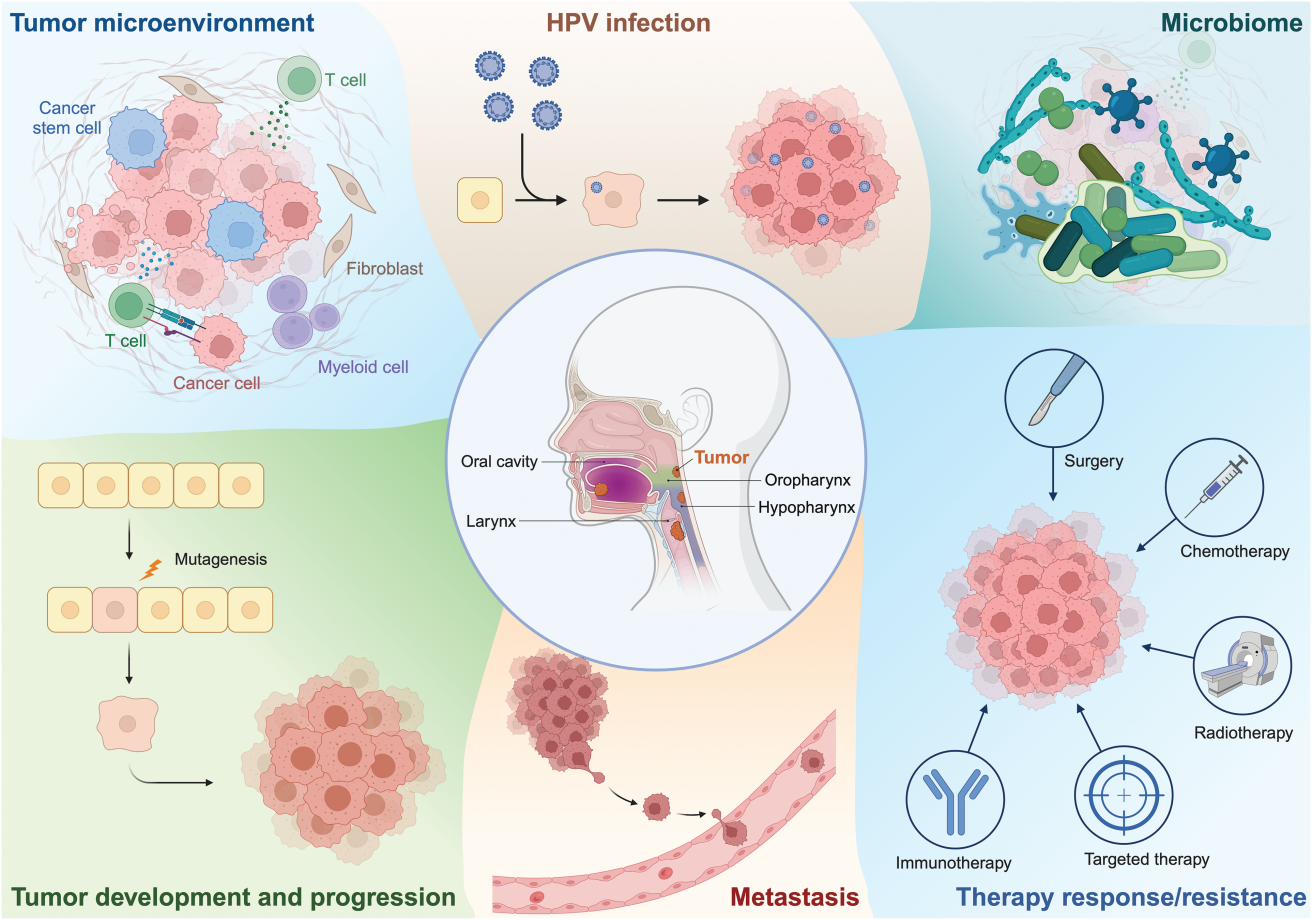
Outline of key topics of this review, encompassing the application of scRNAseq and spatial transcriptomics to explore various aspects of HNSCC biology. Figure created with BioRender.com.

A multitude of studies has explored the mutational basis of HNSCC, the deregulation of gene expression and signaling pathways, and the composition and functions of the tumor microenvironment (TME), with the goal of developing biomarkers for therapy responsiveness as well as novel drug targets, yet, as described above, so far with limited clinical impact. Among the key impediments to the translation of oncological research results into therapeutic applications are intra-tumoral heterogeneity (ITH), i.e., the composition of tumors of cells with different patterns of genetic and transcriptional aberrations, and plasticity, i.e., the ability of tumor cells to change between different transcriptional and functional states [22–24]. In recent years, methods allowing the assessment of gene expression patterns of (dissociated) single cells, or of small groups of cells in their original spatial context, have been developed and greatly improved the resolution of studies exploring ITH, the TME, and intra-tumoral cell-cell communication. This review summarizes what has been learned from the application of such advanced omics techniques to the investigation of HNSCC (Fig. 1 and Table 1).

**Table 1:** Summary of the scRNAseq datasets discussed in this review

		Donors		Sex			Age	HPV status			Samples									
Sub-entity^a^	scRNAseq method	Patients	Healthy	m	f	NA	Years (median)	Pos	Neg	NA	Cell selection	Tumor	LN	OLK	Adj normal	PBMC HNSCC	PBMC HD	Healthy tonsil	Captured cells (No.)	Ref.
OSCC	SMART-Seq2	18	0	9	9	0	70	0	10	8	none	18	5	–	–	–	–	–	5,901	[82]
LSCC	Rhapsody™ system	2	0	1	1	0	65	0	2	0	none	2	–	–	–	–	–	–	14,411	[84]
HPSCC	Chromium Single Cell 3' v3	2	0	0	0	2	66	0	0	2	none	2	–	–	2	–	–	–	17,599	[85]
OSCC	Chromium Single Cell 3' v2	18	0	13	5	0	NA	6	12	0	CD45^−^	15	–	–	–	18	–	–	134,606	[86]
LSCC											CD45^+^	18								
OPSCC																				
OSCC	Chromium Single Cell 3' v3	3	0	2	1	0	56	0	0	3	Calcein^+^ / CD3^+^	3	–	–	3	–	–	–	11,866	[92]
OSCC	Chromium Single Cell 3' v2	26	11	28	9	0	45.2^b,c^	8	18	11 (HD)	CD45^+^	26	–	–	–	–	6	5	131,224	[93]
LSCC							60.3^b,d^													
OPSCC																				
OPSCC^e^	Chromium Single Cell 5'	10	0	8	2	0	64.7^b^	4	6	0	none	2	–	–	5	–	–	–	49,813	[94]
											CD3^+^	7								
OSCC	SMART-Seq2	8	2	6	2	2 (HD)	59.5	3	4	1	lin^−^ / CD45^+^ / CD56^+^ and/or CD127^+^	8	4	–	–	4	2	–	2,747	[98]
OPSCC																				
OPSCC	Chromium Single Cell 3'	16	0	14	2	0	61.5	11	5	0	none	12	1	–	3	–	–	–	70,970	[99]
	Chromium Single Cell 5'										CD45^−^	4								
											CD45^+^	4								
OSCC	Chromium Single Cell 3' v2	23	0	17	6	0	58	13	24	0	none	20	4	4	9	–	–	–	54,239	[100]
OPSCC																				
HPSCC																				
HNSCC	Rhapsody™ system	1	0	0	0	1	NA	0	1	0	none	1	–	–	–	–	–	–	NA	[103]
HNSCC	Chromium Single Cell 5'	12	0	0	0	12	NA	12	0	0	CD8^+^	10	9	–	–	–	–	–	NA	[104]
OPSCC	Chromium Single Cell 3' v3.1	55	0	46	9	0	56	37	18	0	none	28	–	–	–	–	–	–	17,315	[105]
OSCC	Chromium Single Cell 3' v3.1	5	0	3	2	0	64	0	0	2	none	5	–	4	4	–	–	–	52,721	[108]
OSCC^f^	Chromium Single Cell 3'	17	0	11	6	0	54	0	0	17	none	13	–	3	8	–	–	–	131,702	[109]
OLK	Chromium Single Cell 5'																			
HNSCC	C1™ Single-Cell Reagent	7	0	0	0	7	21-85^g^	0	7	0	none	7	7	–	–	–	–	–	53,459	[110]
HNSCC	Chromium Single Cell 3'	4	0	0	0	4	NA	0	0	8	none	8	–	–	–	–	–	–	22,906	[114]
NPSCC → LSCC	Chromium Single Cell V(D)J v1.1	1	0	1	0	0	50	0	0	3	none	2	1	–	–	–	–	–	11,470	[115]
OSCC	Chromium Single Cell 5' v1	27	0	18	11	0	62^b^	0	6	21	none	25	–	–	–	27	–	–	74,557	[116]
											CD45^+^					6				
											CD4^+^									
											CD8^+^									
																6				
HNSCC	Chromium Single Cell 3' v2	6	0	2	4	0	66.5	0	6	0	CD3^+^	6	–	–	–	–	–	–	33,190	[117]
	Chromium Single Cell 5' v1.1																			
HPSCC	Chromium single cell^h^	8	0	8	0	0	57	0	0	8	none	12	1	–	2	–	–	–	89,094	[118]
											CD45^−^	1								
											CD45^+^	1								
OSCC	Chromium Single Cell 3' v3.1	3	0	3	0	0	NA	0	0	3	none	3	–	–	–	–	–	–	3,369	[119]
HNSCC	Chromium Single Cell 3' v2	4	0	1	3	0	71	0	0	4	none	4	–	–	–	–	–	–	NA	[127]

Note: Chromium series, Rapsody, and C1^™^ are from 10x Genomics, BD, and Fluidigm, respectively. Adj, adjacent; f, female; HD, healthy donor; HNSCC, head and neck squamous cell carcinoma; HPSCC, hypopharyngeal squamous cell carcinoma; LN, lymph node metastases; LSCC, laryngeal squamous cell carcinoma; NPSCC, nasopharyngeal squamous cell carcinoma; m, male; NA, not available; OLK, oral leukoplakia; OPSCC, oropharyngeal squamous cell carcinoma; OSCC, oral squamous cell carcinoma; PBMC, peripheral blood mononuclear cells; Ref., reference. ^a^HNSCC if subentity not provided, ^b^mean, ^c^healthy donors, ^d^HNSCC patients, ^e^mainly OPSCC, ^f^from one OSCC patient, only normal adjacent tissue was available, ^g^range, ^h^no details provided.

## scRNAseq and Spatial Transcriptomics: A Methods Overview

In modern translational oncological research, genome-wide screens aiming to identify either genetic alterations like mutations or copy number variations (CNVs) or differentially expressed genes have yielded fundamental insights into the molecular landscapes of tumors. At the beginning of the omics era, microarray-based approaches were used for these purposes, but have lost their significance in recent years due to the development of next-generation sequencing methods. Technological advances in analysing whole genomes/exomes, methylomes, and transcriptomes in a time- and cost-effective manner facilitated the establishment of large, publicly accessible repositories hosting genetic, epigenetic, gene expression, and clinical data from thousands of patients with a variety of different tumors, e.g., the Gene Expression Omnibus and The Cancer Genome Atlas (TCGA) data collections [25]. However, bulk next-generation sequencing cannot address in which cell type deregulation of a gene occurs, whether it is uniform in all cells of this type, and whether it is even due to upregulation in a specific cell type or rather reflects a shift in cell type composition. This underscores the importance of single-cell analyses, which offer the ability to capture the spectrum of cellular diversity, identify novel cell types, track developmental lineages, and illuminate the complex interplay of cells within tissues. Spatial methods further enhance this perspective by pinpointing the exact location of these cells within their biological context.

Various technologies have been established to analyze the transcriptome at single-cell resolution, including Drop-seq [26], the Chromium platform (10× Genomics) [27,28], Switching Mechanism at 5′ end of RNA Template Sequencing (SMART-seq2) [29], Cell Expression by Linear amplification and Sequencing (CEL-seq) [30], QUARTZ-seq [31], and Massively parallel RNA Single-cell sequencing (MARS-seq) [32]. Each of these technologies has its own advantages and limitations and is suitable for certain applications.

Drop-seq and the 10× Genomics Chromium platform are based on microfluidic technology. The standard 10× single-cell gene expression experiment allows for the analysis of transcriptomes from up to 10,000 single cells (20,000 in a recently released high-throughput variant). In this process, individual cells are enclosed in microscopic droplets of a water-oil emulsion. Each droplet also contains a gel bead carrying the reagents for complementary DNA (cDNA) synthesis, including a primer equipped with a unique barcode. When the cell and the gel bead meet in the emulsion, the cell is lysed and cellular RNA is released. The RNA molecules then hybridize with the primers on the gel bead and are converted into cDNA. Once this step is completed, the droplets are dissolved, followed by cDNA amplification, library preparation and sequencing. The resulting sequencing data contain the individual barcodes, which allow the sequences to be assigned to specific cells using bioinformatic methods.

Unlike the Chromium platform, SMART-seq2 allows the capture of full-length transcripts, making it a valuable technique for studying splice variants and alternative transcripts [29,33]. However, the number of cells examined is significantly lower compared to the 10× Genomics technology. CEL-seq uses linear amplification of mRNA to determine gene expression profiles [30]. With this method, individual cells are isolated, e.g., by microfluidics, and extracted mRNA is bound to primers that contain both an oligo(dT) sequence and a cell-specific barcode sequence. The mRNA is converted into cDNA followed by linear amplification through *in vitro* transcription, which leads to less distortion in gene expression compared to exponential amplification. The resulting RNA serves as input for sequencing library preparation. Other single-cell RNA sequencing (scRNAseq) approaches that also utilize *in vitro* transcription for linear amplification are QUARTZ-seq [31] and MARS-seq [32].

Overall, the choice of scRNAseq method strongly depends on the specific requirements of the experiment. While Chromium and Drop-seq are ideal for large-scale, high-throughput projects, SMART-seq2, CEL-seq and QUARTZ-seq are better suited for applications that require high sensitivity and gene coverage [34]. However, scRNAseq methods fall short of retaining the original spatial positioning of the cells within the tissue context. This gap is filled by spatial transcriptomics, which preserves the spatial information of gene expression and enables the analysis of cellular behavior and interactions within the native tissue architecture [35]. Several methods are available for spatial transcriptomics [36]. In the Visium process (10× Genomics), tissue sections are mounted on a glass slide harbouring a grid of oligonucleotides that contain a sequencing handle needed for PCR amplification, a location-specific barcode, and an oligo(dT) sequence. Tissue sections are permeabilized and RNA is hybridized to probe pairs recognizing adjacent sequences in their respective target transcripts. Bound probe pairs are ligated, captured via the oligo(dT) portion of the array-bound oligonucleotides, amplified, and used for sequencing library preparation [27,28]. In the GeoMx procedure (NanoString), a tissue sample fixed on a glass slide is stained with antibodies (for protein assay) or probes (for RNA assay) that are linked to photocleavable oligonucleotides. Pre-designed assays for targets like immune response proteins or specific transcript sets are available. From digital images of the tissue sample, regions of interest (ROIs) are selected for UV exposure, which prompts the release of oligonucleotides from the antibodies/probes. The subsequent quantification of these oligonucleotides provides a measure of the relative abundance of their corresponding targets within the analyzed ROIs [37].

## Bioinformatics Tools for the Analysis of scRNAseq Data

**Figure 2:**
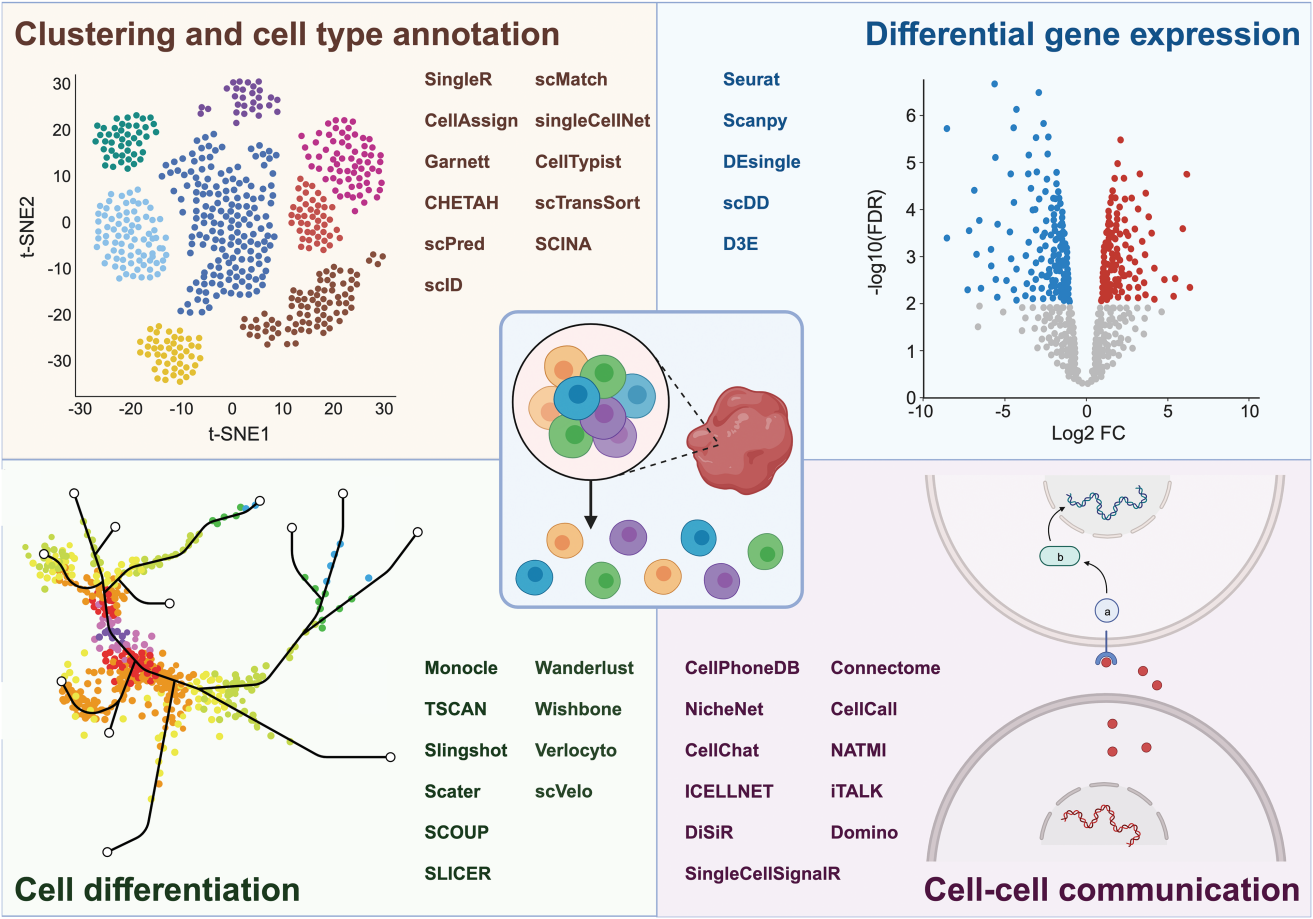
Overview of aspects that can be investigated by scRNAseq, including cell clustering and cell type annotation, differential gene expression, cell differentiation, and cell-cell communication, along with the respective bioinformatic tools used to unravel these processes. Figure created with BioRender.com.

Along with scRNAseq technology, bioinformatics tools to analyze the resulting data have been developed. The R package Seurat and the Python-based tool Scanpy [38–40] both provide functions for quality control, data normalization, clustering, dimension reduction, and data visualization. Various other R packages can be integrated into the Seurat pipeline for the prediction of CNVs, automated cell type annotation, as well as for exploring relationships between cells connected through pseudotime differentiation trajectories. CNVs are a defining feature of tumor cells and can be detected by CAISIC [41], CaSpER [42], or InferCNV [43], which assumes that genes located in regions with CNVs will also display alterations in their RNA expression levels. Several tools are available for cell type annotation [44–57]. SingleR [58] and CellAssign [59] facilitated cell type identification based on reference datasets from pure cell types and known marker gene sets, respectively. Garnett, on the other hand, classifies cells by a user-defined hierarchy of cell types and marker genes for each cell type [60]. Cellular trajectory analysis, or lineage tracing, maps the developmental pathway of a cell along a maturation pathway [61]. Pseudotime analysis, an integral part of this process, provides a way to order cells along these developmental pathways based on incremental alterations of their transcriptional profiles from a starting point (e.g., a naïve T-cell) to an end point (e.g., a cytotoxic T-cell). A variety of computational tools including Monocle [62], TSCAN [63], and Slingshot [64] have been developed to conduct these analyses [65–71] (Fig. 2).

scRNAseq can also be used to develop hypotheses of how cells interact with each other through signaling molecules [72–81]. CellPhoneDB uses a permutation-based statistical method to analyze the potential interactions between cell types based on the average expression of known ligand-receptor pairs and calculates whether any given pair of cells, each expressing either a ligand or the corresponding receptor, are more likely to interact than would be expected by chance [76]. NicheNet constructs an interaction network representing potential cell-cell communications by analyzing ligand production in sender cells, receptor expression in receiver cells, and the predicted downstream genes in the receiver cells [77]. CellChat aims at identifying potential intercellular communication based on signaling pathway activity [78] (Fig. 2).

## Cell Type Composition and Cell-Cell Communication in HNSCC

In the first study to apply scRNAseq to HNSCC, primary tumor samples from 18 treatment-naïve patients with oral squamous cell carcinoma (OSCC) as well as five partially matched lymph node (LN) metastases were investigated [82]. Non-malignant cells formed eight clusters comprising T-cells, B-cells, macrophages, dendritic cells, mast cells, endothelial cells, fibroblasts, and myocytes, respectively. T-cells sub-clustered into conventional helper T-cells (CD4^+^ Tconv), regulatory T-cells (Tregs), CD8^+^ T-cells, and exhausted CD8^+^ T-cells, with the relative size of the latter group varying considerably between patients. Fibroblasts comprised the subclusters myofibroblasts, cancer-associated fibroblasts (CAFs), and resting fibroblasts [82]. In contrast to non-malignant cells, whose respective gene expression patterns were largely consistent across different tumors, malignant cells, identified based on the expression of epithelial markers and the presence of inferred CNVs, clustered in a patient-specific manner. Confirming the heterogeneity of tumor cells, of 60 signatures defining malignant cell subsets, only seven were present in more than one tumor. Specifically, an expression program related to extracellular matrix and epithelial-mesenchymal transition (EMT) was found in 7/10 patients included in this part of the analysis [82]. This signature was referred to as partial EMT, or p-EMT, signature, because expression of most of the key EMT transcription factors was undetectable (yet inferred based on the expression of their target genes in a re-analysis of these data [83]). The p-EMT signature was also expressed in a subset of cells from the OSCC cell line SCC-9 and associated with decreased proliferation and increased invasiveness. Immunohistochemistry showed that cells co-expressing p-EMT markers localized to the leading edge of primary tumors, while cells positive for epithelial differentiation markers were situated in the tumor core. The expression patterns of ligands and receptors, together with *in vitro* experiments, suggested that CAFs signaling through the TGF-β pathway promoted expression of the p-EMT program in cancer cells. High p-EMT scores in bulk RNA expression data from the TCGA cohort were associated with advanced tumor grade, lymphovascular invasion, and LN metastases [82]. Finally, the scRNAseq data, along with deconvolution of bulk expression data from the TCGA HNSCC cohort, confirmed the existence of the previously defined basal, classical, and atypical subtypes of HNSCC, while the mesenchymal subtype was more likely to reflect a higher proportion of fibroblasts than a gene expression program intrinsic to tumor cells [25,82].

Subsequent studies on two patients each with laryngeal squamous cell carcinoma (LSCC) [84] and hypopharyngeal squamous cell carcinoma (HPSCC) [85], as well as on 18 patients with HNSCC from various subsites and with variable HPV status [86], confirmed the presence of the major cell type clusters reported in the above-described OSCC study [82]. Moreover, the pan-HNSCC study identified a fibroblast subcluster expressing elastic fiber differentiation genes, a cell type not previously reported in HNSCC, but confirmed by deconvolution of the TCGA data. The corresponding signature, as well as a signature characterizing CAFs, was associated with shorter survival among HPV^+^ patients within the TCGA cohort [86]. The same study also corroborated the previously reported [82] patient-specific clustering of malignant (keratin-expressing, CNV-bearing) cells, with higher similarity among cells from patients sharing the same HPV status [86]. Similar to the observations in OSCC, keratinocyte-like cells were located in the tumor core of LSCC, and the expression of the respective marker genes correlated with longer survival in the TCGA data set [84]. Proliferative tumor cells, on the other hand, were located at the tumor edge, and the expression of their marker genes correlated positively with tumor grade [84]. In HPSCC, ligand-receptor analyses, immunohistochemistry, and cell culture experiments suggested a tumor-promoting role for the TGF-β family member BMP4, produced by CAFs and activating BMPR2 on cancer cells [85].

Even though not uncontroversially, cancer stem cells are considered as the source of cancer cell renewal and therapy resistance [24,87]. Some studies have therefore attempted to investigate HNSCC stem cells through single-cell transcriptomics. As an example, Johansson *et al* performed scRNAseq on organoids formed by stem cell-containing Sox2^+^ keratinocytes, which had been collected from mice with 4-nitroquinoline-1-oxide-induced oral carcinomas or control mice. This revealed a single cluster with a high StemID score in the control organoid, but several clusters with a substantially lower score in the cancer organoid [88]. In an integrated analysis of the cells from the tumor and control organoid, the stem-like cells from the two types of organoid clustered separately. Genes pertaining to p53 signaling, apoptotic signaling, and cell cycle arrest were up-regulated in the control stem-like cluster, while HIF-1α signaling, glycolysis, extracellular matrix organization, epithelial cell proliferation, and cell motility were enriched among the genes highly expressed in the cancer stem-like cluster [88].

Primary scRNAseq data are usually made publicly available upon publication of their original analysis and provide a rich resource of information that can be used as a basis for, or a complement to, independent investigations. As an example, Xiao et al. [89] focussed on the activity of metabolic programmes in the above described OSCC scRNAseq dataset [82]. Of 80 metabolic pathways, over 70 showed differential activity between different cell types, indicating that metabolic activity was mainly determined by cell type. The largest number of metabolic pathways was upregulated in malignant cells. Also, CAFs were more metabolically active than myofibroblasts, featuring enhanced glycolysis and production of inflammatory mediators [89]. Principal component analysis followed by gene set enrichment analysis revealed that mitochondrial activity, represented by oxidative phosphorylation and the tricarboxylic acid cycle, was the main determinant of metabolic heterogeneity between malignant cells. Glycolysis, oxidative phosphorylation, and hypoxia signatures were significantly correlated to each other in both malignant and non-malignant cells, suggesting that tumor-related metabolic reprogramming does not consist of a switch between glycolysis and mitochondrial respiration, but rather, oxygen deprivation up-regulates both of these processes, potentially fostering competition of cells for limited resources [89].

In another high-profile study based on the reuse of publicly available scRNAseq data, ITH was investigated in 1,163 tumor samples from 77 different studies representing 24 tumor types [90]. From 5,547 transcriptional programs that varied between malignant cells of individual tumors, 41 meta-programs characterizing ITH were distilled. ITH of seven meta-programs, related to cell cycle, stress, hypoxia, interferon responses, EMT, and MYC targets, was frequent in most cancer types, 21 meta-programs were shared only between a subset of cancer types, and the variability of the remaining meta-programs was tumor-type specific [90]. Interestingly, most malignant meta-programs resembled meta-programs of non-malignant epithelial cells, suggesting that transcriptional ITH reflects the heterogeneity of the tissue from which the tumors emerged [90].

## The Tumor Immune Microenvironment

The immune system is one of the major lines of defense of the human body against malignant cells. Accordingly, immunotherapy, e.g., through modified immune cells or through ICI-mediated re-activation of endogenous immune responses muted by the tumor, has advanced to become one of the most successful therapeutic strategies in oncology [91]. Nevertheless, and albeit approved for HNSCC, ICI is successful only in a minority of patients with this cancer type [16,20,21]. Therefore, several articles have specifically investigated the HNSCC tumor immune microenvironment through scRNAseq on sorted cell populations, e.g., CD45^+^ hematopoietic cells or CD3^+^ T-cells. Some studies included adjacent normal tissue, peripheral blood leukocytes, and/or non-tumorous tonsils in addition to tumor tissue.

Due to their key role in antitumor immunity and immunotherapy, many analyses focussed on (cytotoxic) T-cells. The T-cell population of tumor tissue contained a substantially higher proportion of CD8^+^ cells than that of adjacent normal tissue (71 *vs*. 52%) [92]. A smooth differentiation trajectory connected peripheral blood (PB)-derived to tumor infiltrating CD8^+^ T-cells, but CD8^+^ T-cells from both PB and non-malignant tonsils were present in different subclusters from their tumor infiltrating counterparts [93]. Naïve-like, cytotoxic, pre-dysfunctional, terminally dysfunctional/exhausted, and cycling cells were identified as major CD8^+^ T-cell subsets [86,94], and all except for the naïve-like subsets were present in higher proportions in the TME compared to adjacent normal tissue and PB [92–95]. Single-cell T-cell receptor sequencing (scTCRseq) further revealed that expanded clonotypes (defined by a clonal size of at least two, and up to 162 cells) were more frequent within tumors than in adjacent normal mucosa, and most abundant among exhausted T-cells [94]. Enhanced expression of the exhaustion marker PD-1 in OSCC-infiltrating *vs*. normal-tissue lymphocytes was confirmed by immunohistochemistry. The transcription factor thymocyte selection-associated high-mobility group box (TOX) was identified as potential regulator of immune checkpoint genes based on scRNAseq data, and its experimental overexpression in primary CD8^+^ T-cells indeed increased their expression of checkpoint genes and diminished their proliferation and cytotoxic activity towards PD-L1 positive HNSCC cell lines *in vitro* [92].

Within the CD4^+^ T-cell population, Tregs were strongly enriched in tumors compared to adjacent normal tissue, further corroborating the immunosuppressive nature of the TME [92–95]. Correspondingly, signatures associated with exhausted CD8^+^ cells and with CD4^+^ Tregs were associated with shorter OS in the TCGA HNSCC data set [92,95].

Evaluation of potential routes of cell-cell communication revealed a massive increase of putative receptor-ligand interactions among tumor infiltrating leukocytes (TILs) *vs*. hematopoietic cells from PB and non-malignant tonsils [93]. Furthermore, macrophages were predicted to represent the main source of PD-L1 for interaction with PD-1 on CD8^+^ T-cells in eight of twelve patients, and epithelial cells in only two of them. Flow cytometry and multispectral fluorescence microscopy confirmed high PD-L1 expression on macrophages as well as their apposition to T-cells. This supported clinical observations that ICI responsiveness was best predicted by a combined PD-L1 score considering both tumor cells and macrophages [16,86].

Woolaver et al. developed a mouse model reflecting the variability of immune responses towards HNSCC: orthotopic (intrabuccal) transplantation of the *Kras*^G12D^ *Smad4*^−/−^ SCC cell line A223 into wildtype C57BL/6 mice initially led to tumor growth in all animals, but was followed by spontaneous regression in 20%–30% of them [96]. CD8^+^ T-cells in regressing tumors were more abundant, more activated, and less exhausted than those in progressing tumors. Supporting the key role of cytotoxic T-cells in the spontaneous regression of tumors in this model, CD8^−/−^ mice were unable to eradicate such tumors, and regressor mice re-challenged with A223 cells cleared tumors rapidly and efficiently [96]. scTCRseq revealed clonal expansion among TILs, but not spleen cells, from both progressors and regressors, and few TCR clonotypes that were shared between samples, suggesting tumor antigen recognition in all recipients, and a highly individualized immune response to these tumors despite their limited genetic heterogeneity [96]. In combined V(D)J- and scRNAseq experiments on CD8^+^ T-cells from three progressors and three regressors, spleen cells dominated the naïve, and TILs the activated cell clusters. TILs from regressors appeared more activated than those from progressors. Specific analysis of the top clonotypes confirmed better activation, stronger cytotoxic ability, and more memory-like states in regressors *vs*. progressors [96].

Hypothesizing that the humoral arm of anti-tumor immunity could offer therapeutic opportunities with the potential to complement current CD8^+^ T-cell focussed immunotherapies, Ruffin et al. focused their analyses on B- and CD4^+^ Tconv cells [97]. scRNAseq data from CD45^+^ cells from the tumors and PB of 27 patients with HNSCC of variable HPV status and from healthy tonsils (in part from the public domain [93]) uncovered 10 clusters of CD4^+^ Tconv cells and 11 clusters of B-cells. Two of the latter were formed by germinal center (GC) B-cells, displaying overlap between TIL-Bs and B-cells from healthy tonsils. In the TCGA dataset, both a high B-cell infiltrate and a high enrichment for GC B-cells were associated with longer progression-free survival. Immunohistochemistry demonstrated that B-cells predominantly infiltrated tertiary lymphoid structures (TLS), which were enriched in HPV^+^ compared to HPV^−^ HNSCC. The numbers of CD4^+^ T-cells and TIL-Bs in TLS were strongly correlated, and in TLS with GC, but not in TLS without GC, TIL-Bs interacted with each other and with CD4^+^ Tconv, suggesting that TLS with GCs play a key role in humoral anti-tumor immunity. Accordingly, intra-tumoral TLS with GC correlated with increased survival in HNSCC [97].

To address the controversial contribution of natural killer (NK) cells to tumor control, Moreno-Nieves et al. performed scRNAseq on innate lymphoid cells (ILCs), which they isolated as CD56^+^ and/or CD127^+^ cells from the primary tumors, LN metastases, and PB of eight HNSCC patients [98]. Among intra-tumoral NK-related cell subsets, intraephithelial ILC cluster 1 (ieILC1) had the greatest cytolytic activity, while NK-2 cells were dysfunctional according to gene expression patterns. Pseudotime analysis suggested that these two cell types represented the end points of two different differentiation trajectories of circulating NK cells. In coculture with HNSCC cells, IL-15 and TGF-β promoted upregulation of the ieILC1 markers CD49a and CD103 in a subset of circulating NK cells, while cells that remained CD49a^−^ resembled NK-2 cells. In response to tumor cells or other stimuli, *in vitro* differentiated ieILC1-like cells degranulated more, produced more interferon γ and tumor necrosis factor α, and killed tumor cells more effectively than NK-2 like cells. Moreover, upon subcutaneous co-injection with HNSCC cells into immunocompromised mice, *in vitro* differentiated CD49a^+^ cells controlled tumor growth significantly more effectively than their CD49a^−^ counterparts. Thus, among ILC/NK cells in the TME, ieILC1 cells were suggested to have the greatest antitumor activity [98].

## Differences between HPV^−^ and HPV^+^ HNSCC Deciphered by scRNAseq

HPV infection is one of the major risk factors for the development of HNSCC [4]. Even though the HPV-16 E6 and E7 proteins are essential for maintaining malignancy, therapies targeting these proteins have achieved only modest success, indicating a need for improvement [4]. In a recent study, Puram et al. [99] discovered remarkable diversity in OPSCC concerning both CNVs and HPV gene expression, indicating an HPV-induced genomic instability. They and others noted significant variability in HPV expression across different tumors and even within genetic subclones of the same tumor [86,99]. Intriguingly, a subset of cells within HPV^+^ tumors, termed HPVoff, displayed no detectable HPV expression but nevertheless showed high levels of the surrogate marker CDKN2A (p16). HPVoff cells were associated with decreased cell cycle anomalies and decreased evasion from senescence, potentially leading to increased treatment resistance and invasion capabilities. They may resume growth and reactivate HPV expression post-treatment, thus representing key players in recurrent HPV^+^ tumors [99].

By investigating associations between HPV and the TME, HPV^+^ tumors were found to contain larger proportions of NK/T-cells and GC TIL-B cells, but a diminished presence of Tregs, fibroblasts and macrophages, as compared to HPV^−^ tumors [86,93,97,100–103]. Eberhardt et al. uncovered not only a high fraction of HPV-specific CD8^+^ T-cells in the TME, but among them also a unique subset of stem-like cells with considerable proliferative potential [104]. Distinguished by the concurrent expression of the inhibitory receptor PD-1 and the transcription factor TCF-1, these stem-like CD8^+^ T-cells triggered proliferation of effector-like T-cells in response to PD-1 pathway disruption in preclinical models [104]. Alongside these findings, another HPV-specific CD8^+^ T-cell subset characterized by the expression of the NK cell marker CD161 was characterized [105]. Jiang et al. identified a unique subpopulation of macrophages, TCR^+^ macrophages, in both HPV^−^ and HPV^+^ HNSCCs [102]. These macrophages were associated with enhanced phagocytosis and potentially improved prognosis of HNSCC patients, but their functionality may be limited in HPV^−^ HNSCC due to immune response inactivation [102].

Using a 3D organotypic epithelial raft model, Bedard et al. discovered a keratinocyte subpopulation that was expanded and reprogrammed by HPV [106]. These cells (termed HPV-induced differentiation-dissonant epithelial nonconventional cells; HIDDEN) formed a distinct compartment in the infected epithelium and persisted from neoplastic lesions to outright cancer. ELF3/ESE-1 was identified as a key regulator whose depletion in HPV^+^ epithelium greatly reduced HIDDEN cell biomarker expression and compartment formation [106].

## Advanced Omics Methods to Investigate the Role of the Oral Microbiota in OSCC

In addition to viral infection, bacterial and fungal microbiota play a role in the development and progression of HSNCC through production of mutagenic substances, modulation of inflammation and immune responses, and the promotion of epithelial cell proliferation and EMT [6]. Therefore, advanced omics methods have been employed to deepen the understanding of the role of the oral microbiota in HNSCC. GeoMx digital spatial profiling of 77 proteins associated with anti-tumor immunity in eight OSCC samples revealed that bacteria resided in microniches characterized by immunosuppression, reduced vascularization, and a highly transformed phenotype [107]. To be able to simultaneously assess the expression of human genes and the presence of bacterial genera at the single-cell level, invasion-adhesion-directed expression sequencing (INVADEseq) was developed by including a primer targeting a conserved region of bacterial 16S rRNA in the 10× Genomics 5′ library preparation protocol. Control experiments with human cancer cell lines revealed that bacterial infection induced not only transcriptional programmes related to anti-bacterial immune responses, but also EMT- and metastasis-promoting genes [107]. Application of INVADEseq to fresh tumor tissue from seven patients with OSCC indicated that the intra-tumoral microbiota was dominated by the genera *Fusobacterium* and *Treponema*, which were primarily associated with aneuploid epithelial cells highly expressing cancer progression-related signaling pathways, and with monocyte-derived macrophages. Thus, cell-associated intra-tumoral microbiota could drive inter-cellular heterogeneity within immune and epithelial cell populations, and enhance the malignancy of tumor cells [107].

## Tumor Development, Progression, and Metastasis

The development and progression of HNSCC was investigated by scRNAseq of normal tissue, precancerous oral leukoplakia, primary tumors, and metastatic tumors from lymph nodes, gathered from a total of 23 patients [100]. Some epithelial cells in leukoplakia showed CNVs as well as expression of various tumor-related genes similar to malignant cells, suggesting that CNV-driven expression of *TP63* and *ATP1B3* in leukoplakia plays a critical role in the progression to HNSCC [100]. Another study that examined matched normal, dysplastic, and tumor cells from OSCC biopsies observed a gradual increase in the expression of genes involved in pathways such as EMT and mTORC1 [108]. Notably, the study also identified VEGFA as a cancer initiating factor [108]. Moreover, various subtypes of fibroblasts were described to support malignant transformation [100,108,109]. Trajectory analyses showed that fibroblasts in leukoplakia were closer to those in cancerous tissue than those in normal tissue, indicating that they may already have acquired CAF features [100]. Collagen (specifically COL1A1) from fibroblasts induced the expression of *LAIR2* (CD306), a soluble collagen receptor that activates pro-inflammatory processes, in leukoplakia Tregs, thus creating a favorable microenvironment for tumor progression [100]. Further, COL1A1-CD44 and LGALS7B-CXCL8 ligand-receptor interactions between fibroblasts and malignant cells were identified as factors that promote HNSCC progression [100].

By examining seven pairs of primary tumors and cervical LN metastases from HNSCC patients using scRNAseq and scTCRseq and trajectory analysis, a distinct subpopulation of pre-metastatic cells, driven by actionable pathways including AXL and AURK, was uncovered [110]. In addition, a subpopulation within the primary tumor exhibiting high EMT scores, deregulated oxidative phosphorylation, and immune evasion was found. In the TCGA dataset, patients with similar gene expression profiles had significantly poorer outcomes [110]. CD8^+^ T-cell clones displayed different trajectories leading to *SOX4*-mediated T-cell dysfunction, with one path showing a progressive loss of naïve markers and gain of dysfunctional markers [110].

## Treatment Resistance

Primary and secondary resistance occurs with all forms of therapy and is the main reason of cancer-related death [23,111]. Thus, a comprehensive understanding of the underlying mechanisms is crucial for improving treatment strategies and prolonging survival of cancer patients. In this context, single-cell analyses were performed to elucidate how cells react to chemotherapy, radiotherapy, targeted therapy, and ICI therapy in HNSCC [94,112–120].

Osman et al. investigated cisplatin resistance in a human HNSCC cell line by scRNAseq and found p53 signaling, cell cycle, senescence, platinum resistance, and FoxO signaling to be activated in cisplatin resistant cells [112]. Further, an epigenetically primed subpopulation, driven by NRF2, a protein involved in regulation of anti-oxidative stress response and epigenomic changes, was identified, potentially linking cellular oxidative stress response and cisplatin resistance [112]. Among eight patients with HPSCC, responsiveness to a therapy comprising taxol, cisplatin, 5-fluorouracil, and cetuximab was associated with a higher number of infiltrating immune cells both before and after therapy as determined by scRNAseq and multiplex immunohistochemistry [118]. Using a cell type deconvolution strategy on a bulk RNAseq dataset from 44 patients, along with classifier training, treatment response could be predicted based on sets of non-malignant cell subtypes that were categorized as either “tumor-suppressive” or “tumor-promoting” [118].

Utilizing a 31-gene signature reflecting radiation sensitivity in a cell line model, Li et al. re-analyzed a publicly available scRNAseq dataset to study the heterogeneity of sensitivity to radiation in OSCC cells [82,121]. The atypical subtype was associated with sensitivity to radiation, and within the classical and basal subtypes gene co-expression modules related to radioresistance (primarily cell division and cell cycle regulation) were identified [121]. Moreover, increased immune checkpoint interactions were seen in radioresistant tumors, theoretically suggesting a basis for combining radiotherapy and immune checkpoint blockade in treating HNSCC. However, this approach has so far not proven successful in clinical studies [15–17].

CDK4/6 inhibitors have been approved for the treatment of breast cancer and are under consideration for a variety of other tumors [122]. In HNSCC, promising results were seen when administering CDK4 inhibitors to HPV^−^, but not HPV^+^ patients [123]. Cheng et al. analyzed 14 paired HNSCC and adjacent normal samples and discovered a proliferative exhausted CD8^+^ T-cell (P-Tex) cluster, beneficial to the survival outcomes of HPV^+^ HNSCC patients [94]. CDK4 levels in these cells were as high as in cancer cells, suggesting that treatment failure in HPV^+^ HNSCC patients could be due to the unintended inhibition of beneficial P-Tex cells by CDK4 inhibitors.

Several studies utilized scRNAseq to analyze response to ICI therapy. In mouse models, successful anti-PD-1 or anti-CTLA-4 therapy enhanced the differentiation of T-cells into more activated states [113]. ICI-resistant tumors were infiltrated with high numbers of tumor associated macrophages (particularly of the immunosuppressive M2 type) supported by expression of CSF1 and VEGF by tumor cells [113,124]. In the human context, Obradovic et al. assessed whether CAF-related or other TME subpopulations may regulate clinical responses to nivolumab by scRNAseq of four tumors from patients with advanced-stage HNSCC before and after ICI treatment [114]. Of five distinct CAF clusters, two emerged as predictive of nivolumab response, while a third was associated with immunosuppression [114].

A recent article provides insights into the role of TILs, specifically CD8^+^ T-cells exhibiting a tissue-residency program (Trm), in the response to neoadjuvant ICI through scRNAseq and scTCRseq on TILs from 19 OSCC patients post-treatment, with pre-treatment biopsies available for six of them [116]. Trm cells, defined by the markers CD103 and HOBIT, were early responders to ICI. They displayed gene expression patterns related to activation, cytotoxicity, and effector function, but also inhibitory receptors including PD-1, proposed to reflect regulation rather than exhaustion. Most TCR clonotypes that expanded during treatment were already present in the tumor prior to treatment, while some became detectable only after treatment. Thus, early intra-tumoral ICI responses may be primarily mediated by pre-existing T-cells with a Trm gene program, while new T-cell populations may be primed elsewhere and then enter the tumor. Moreover, a high proportion of PD-1^+^ KLRG1- CD8^+^ T-cells in PB correlated strongly with responsiveness to neoadjuvant ICI therapy, indicating the potential usefulness of these cells as a predictive biomarker in this context [116]. Another study characterized CD4^+^ and CD8^+^ T-cell dynamics by scRNAseq of TILs isolated from tumor biopsies of six HNSCC patients before and after neoadjuvant treatment with bintrafusp alfa, a bifunctional fusion protein consisting of a monoclonal anti-PD-L1 antibody and a TGF-β receptor extracellular domain capable of concurrently blocking PD-1 signaling and neutralizing TGF-β in the TME [117]. This therapy predominantly activated exhausted CD8^+^ TILs, which was associated with altered glutamine metabolism. Abrogation of TGF-β signaling was suggested to promote the egress of exhausted TILs to the blood after treatment, which could improve patient outcomes [117].

Lin et al. examined a unique patient who developed three separate HNSCCs, two of which were responsive to ICI treatment, while the third progressed under therapy [115]. The T-cell exhaustion markers, *LAG3* and *PTPN6*, were expressed at higher levels in the exhausted CD8^+^ T-cells of the progressing lesion as compared to the responding ones, suggesting the upregulation of alternative inhibitory receptors in exhausted CD8^+^ T-cells as a potential mechanism of ICI resistance [115].

## Insights from Large-Scale Gene Expression Profiling with Spatial Resolution

Being able to analyze the gene expression patterns of single cells has substantially advanced our understanding of tumor biology. However, since the scRNAseq workflow is based on isolation of dissipated cells, spatial information is lost. The development of methods preserving this information is rapidly advancing, and some examples of their application to HNSCC are described below.

To investigate differences in gene expression patterns between more broadly defined tumor areas, Chung *et al* performed bulk RNAseq on specimens from the inner core and invasive front of primary tumors, as well as from metastases and adjacent normal mucosa from 21 patients with HNSCC. Progressive enrichment of signatures associated with EMT, inflammation, and ferroptosis was observed from inner core to invasive front and metastatic samples [119]. scRNAseq on three, and spatial transcriptomics via the 10× Genomics Visium platform on two primary HNSCC samples confirmed and refined these data on the single-cell level and at high spatial resolution, respectively [119]. Intersection of the genes associated with a high ferroptosis score in the primary samples with those induced by a sublethal dose of the ferroptosis inducer FIN56 in an HNSCC cell line revealed *CD274*, encoding PD-L1, as the central hub gene [119]. Correspondingly, FIN56 induced PD-L1, and cooperated with PD-L1 antibody to suppress tumor formation, in a syngeneic HNSCC mouse model [119].

Stimulated Raman scattering microscopy exploits differences between the vibrations of CH_2_ and CH_3_ bonds and the differential abundance of such bonds in lipids and proteins to provide detailed information on tissue architecture in fresh frozen tissue slices. Increasing the intensity of the excitation source facilitates the excision of ROIs, which can be subjected to parallel extraction of high quality DNA and RNA [125]. In a proof-of-principle study including samples from four patients with OSCC, from which ROIs containing ~230 cells were excised, novel gene fusions were identified, and both DNA-derived CNV data and transcriptomic data revealed large interpatient variability, and even variability between different cancer nests from the same patient [125].

Another major line of technical development concerns the facilitation of simultaneous detection of multiple proteins through immunological methods. As an example, CO-DEtection by indeXing (CODEX) permits visualization of up to 60 markers through DNA-conjugated antibodies that are sequentially detected via complementary, fluorescently labeled DNA probes [126]. To simplify and objectify interpretation of the resulting data, Zhang et al. developed cell type identification with spatial information (CELESTA), an unsupervised machine learning algorithm that assigns cell types based on the expression of marker proteins and on spatial information, and applied it to CODEX imaging with 52 markers of samples from each four HNSCC patients with and without LN metastases [127]. Malignant cells and Tregs were found to colocalize more often in samples with than without LN involvement. This was confirmed by staining for FOXP3 (Tregs) and cytokeratin (malignant cells) on a tissue microarray from an independent patient cohort. scRNAseq on four samples suggested that interactions between CXCL10 and CXCR3, expressed on malignant cells and Tregs, respectively, may mediate their colocalization. Indeed, cancer cells with higher CXCL10 expression more effectively stimulated migration of CXCR3^+^ Tregs in a transwell experiment, and the CXCR3 inhibitor AMG487 reduced the number of Tregs recruited to tumors in a mouse model [127].

Even though studied to a much lesser extent than TILs and CAFs, nerves represent an important component of the TME [128]. In HNSCC and other tumors, they play roles in cancer-associated pain and aggressiveness. Tumors recruit nerves, which in turn foster the proliferation of malignant cells. These may even invade the nerve structure, a phenomenon called perineural invasion (PNI) [128]. Through re-analysis of publicly available scRNAseq data [82], a gene expression module associated with PNI in the TCGA dataset was found to correlate positively with EMT, metastasis, and invasion, and negatively with stemness, in malignant cells from HNSCC samples [129]. Using immunohistochemistry for the neuronal marker protein S100, Schmitd et al. detected PNI in 43% of 142 patients with OSCC [130]. PNI was more prevalent in advanced stage, larger tumors, and tumors with LN metastases. Among PNI^+^ patients, the distance between nerve and tumor cells was predictive of disease-free, disease-specific, and overall survival [130]. Samples from eight patients with OSCC were subjected to whole transcriptome expression profiling using the NanoString GeoMx Digital Spatial Profiler platform. Of 8,162 genes whose expression could be detected, 95 were up- and 64 down-regulated in nerves close to tumor (NC; <100 µM distance and 50% of the nerve circumference surrounded by tumor cells) *vs*. nerves far from tumor (NF; >1 mm distance) [130]. Pathway enrichment analysis of the differentially expressed genes suggested that NC are exposed to stress exerted by the tumor and undergo degenerative processes. However, differential expression of individual genes indicated that NC may also undergo reprogramming to survival mode and increase myelination to support nerve regeneration [130].

## Concluding Remarks and Future Perspectives

Single-cell sequencing approaches represent a significant addition to the scientific toolbox, enabling a comprehensive understanding of tumor complexity, evolution, and interactions between tumor cells and their microenvironment. Despite its considerable advantages, scRNAseq faces several challenges including costs, technical hurdles during isolation of single cells without damage, data complexity, addressing errors and biases within data, and the loss of spatial context when removing a cell from tissue. The latter issue is addressed by spatial transcriptomics, however, improvements such as enhancing gene coverage—which also needs refinement in scRNAseq—and spatial resolution are necessary. In conclusion, while scRNAseq offers significant advancements over traditional methods like immunohistochemistry or bulk transcriptomics approaches by enabling an in-depth analysis of individual cells, it is still a developing field with notable challenges. Its potential to open new avenues to deeper understand tumor biology makes it an essential tool for future medical and scientific breakthroughs.

## Data Availability

Not applicable.

## References

[1] Cook, M., Dawsey, S., Freedman, N., Inskip, P., Wichner, S. et al. (2009). Sex disparities in cancer incidence by period and age. Cancer Epidemiology, Biomarkers & Prevention*,* 18 *(*4*),* 1174–1182. 10.1158/1055-9965.EPI-08-1118; 19293308 PMC2793271

[2] Gormley, M., Creaney, G., Schache, A., Ingarfield, K., Conway, D. (2022). Reviewing the epidemiology of head and neck cancer: Definitions, trends and risk factors. British Dental Journal*,* 233 *(*9*),* 780–786. 10.1038/s41415-022-5166-x; 36369568 PMC9652141

[3] Hashibe, M., Brennan, P., Chuang, S. C., Boccia, S., Castellsague, X. et al. (2009). Interaction between tobacco and alcohol use and the risk of head and neck cancer: Pooled analysis in the International Head and Neck Cancer Epidemiology Consortium. Cancer Epidemiology, Biomarkers & Prevention*,* 18 *(*2*),* 541–550. 10.1158/1055-9965.EPI-08-0347; 19190158 PMC3051410

[4] Santegoets, S. J., Welters, M. J. P., Schrikkema, D. S., Freriks, M. R., Kok, H. et al. (2023). The common HLA class I-restricted tumor-infiltrating T cell response in HPV16-induced cancer. Cancer Immunology and Immunotherapy*,* 72 *(*6*),* 1553–1565. 10.1007/s00262-022-03350-x; 36526910 PMC10198845

[5] Burcher, K., Burcher, J., Inscore, L., Bloomer, C., Furdui, C. et al. (2022). A review of the role of oral microbiome in the development, detection, and management of head and neck squamous cell cancers. Cancers*,* 14 *(*17*),* 4116. 10.3390/cancers14174116; 36077651 PMC9454796

[6] Stasiewicz, M., Karpinski, T. (2022). The oral microbiota and its role in carcinogenesis. Seminars in Cancer Biology*,* 86 *(*3*),* 633–642. 10.1016/j.semcancer.2021.11.002; 34743032

[7] Lechner, M., Liu, J., Masterson, L., Fenton, T. (2022). HPV-associated oropharyngeal cancer: Epidemiology, molecular biology and clinical management. Nature Reviews Clinical Oncology*,* 19 *(*5*),* 306–327. 10.1038/s41571-022-00603-7; 35105976 PMC8805140

[8] Sharkey Ochoa, I., O'Regan, E., Toner, M., Kay, E., Faul, P. et al. (2022). The role of HPV in determining treatment, survival, and prognosis of head and neck squamous cell carcinoma. Cancers*,* 14 *(*17*),* 4321. 10.3390/cancers14174321; 36077856 PMC9454666

[9] Machiels, J., Rene Leemans, C., Golusinski, W., Grau, C., Licitra, L. et al. (2020). Squamous cell carcinoma of the oral cavity, larynx, oropharynx and hypopharynx: EHNS-ESMO-ESTRO clinical practice guidelines for diagnosis, treatment and follow-up. Annals of Oncology*,* 31 *(*11*),* 1462–1475. 10.1016/j.annonc.2020.07.011; 33239190

[10] Leemans, C., Tiwari, R., Nauta, J., van der Waal, I., Snow, G. (1994). Recurrence at the primary site in head and neck cancer and the significance of neck lymph node metastases as a prognostic factor. Cancer*,* 73 *(*1*),* 187–190. 10.1002/1097-0142(19940101)73:1<187::aid-cncr2820730132>3.0.co;2-j; 8275423

[11] Sturgis, E., Miller, R. (1995). Second primary malignancies in the head and neck cancer patient. The Annals of Otology, Rhinology & Laryngology*,* 104 *(*12*),* 946–954. 10.1177/000348949510401206; 7492066

[12] Bonner, J., Harari, P., Giralt, J., Cohen, R., Jones, C. et al. (2010). Radiotherapy plus cetuximab for locoregionally advanced head and neck cancer: 5-year survival data from a phase 3 randomised trial, and relation between cetuximab-induced rash and survival. Lancet Oncology*,* 11 *(*1*),* 21–28. 10.1016/S1470-2045(09)70311-0; 19897418

[13] Gillison, M., Trotti, A., Harris, J., Eisbruch, A., Harari, P. et al. (2019). Radiotherapy plus cetuximab or cisplatin in human papillomavirus-positive oropharyngeal cancer (NRG Oncology RTOG 1016): A randomised, multicentre, non-inferiority trial. Lancet*,* 393 *(*10166*),* 40–50. 10.1016/S0140-6736(18)32779-X; 30449625 PMC6541928

[14] Gebre-Medhin, M., Brun, E., Engstrom, P., Haugen Cange, H., Hammarstedt-Nordenvall, L. et al. (2021). ARTSCAN III: A randomized phase III study comparing chemoradiotherapy with cisplatin versus cetuximab in patients with locoregionally advanced head and neck squamous cell cancer. Journal of Clinical Oncology*,* 39 *(*1*),* 38–47. 10.1200/JCO.20.02072; 33052757 PMC7771720

[15] Lee, N., Ferris, R., Psyrri, A., Haddad, R., Tahara, M. et al. (2021). Avelumab plus standard-of-care chemoradiotherapy versus chemoradiotherapy alone in patients with locally advanced squamous cell carcinoma of the head and neck: A randomised, double-blind, placebo-controlled, multicentre, phase 3 trial. Lancet Oncology*,* 22 *(*4*),* 450–462. 10.1016/S1470-2045(20)30737-3; 33794205

[16] Machiels, J., Tao, Y., Burtness, B., Tahara, M., Rischin, D. et al. (2022). Primary results of the phase III KEYNOTE-412 study: Pembrolizumab (pembro) with chemoradiation therapy (CRT) vs placebo plus CRT for locally advanced (LA) head and neck squamous cell carcinoma (HNSCC). Annals of Oncology*,* 33 *(*33*),* S1399.

[17] Tao, Y., Biau, J., Sun, X., Sire, C., Martin, L. et al. (2023). Pembrolizumab versus cetuximab concurrent with radiotherapy in patients with locally advanced squamous cell carcinoma of head and neck unfit for cisplatin (GORTEC 2015-01 PembroRad): A multicenter, randomized, phase II trial. Annals of Oncology*,* 34 *(*1*),* 101–110. 10.1016/j.annonc.2022.10.006; 36522816

[18] Uppaluri, R., Campbell, K., Egloff, A., Zolkind, P., Skidmore, Z. et al. (2020). Neoadjuvant and adjuvant pembrolizumab in resectable locally advanced, human papillomavirus-unrelated head and neck cancer: A multicenter, phase II trial. Clinical Cancer Research*,* 26 *(*19*),* 5140–5152. 10.1158/1078-0432.CCR-20-1695; 32665297 PMC7547532

[19] Wise-Draper, T., Gulati, S., Palackdharry, S., Hinrichs, B., Worden, F. et al. (2022). Phase II clinical trial of neoadjuvant and adjuvant pembrolizumab in resectable local-regionally advanced head and neck squamous cell carcinoma. Clinical Cancer Research*,* 28 *(*7*),* 1345–1352. 10.1158/1078-0432.CCR-21-3351; 35338369 PMC8976828

[20] Burtness, B., Harrington, K., Greil, R., Soulieres, D., Tahara, M. et al. (2019). Pembrolizumab alone or with chemotherapy versus cetuximab with chemotherapy for recurrent or metastatic squamous cell carcinoma of the head and neck (KEYNOTE-048): A randomised, open-label, phase 3 study. Lancet*,* 394 *(*10212*),* 1915–1928. 10.1016/S0140-6736(19)32591-7; 31679945

[21] Harrington, K., Burtness, B., Greil, R., Soulieres, D., Tahara, M. et al. (2023). Pembrolizumab with or without chemotherapy in recurrent or metastatic head and neck squamous cell carcinoma: Updated results of the phase III KEYNOTE-048 study. Journal of Clinical Oncology*,* 41 *(*4*),* 790–802. 10.1200/JCO.21.02508; 36219809 PMC9902012

[22] Mroz, E. A., Tward, A. D., Pickering, C. R., Myers, J. N., Ferris, R. L. et al. (2013). High intratumor genetic heterogeneity is related to worse outcome in patients with head and neck squamous cell carcinoma. Cancer*,* 119 *(*16*),* 3034–3042. 10.1002/cncr.28150; 23696076 PMC3735618

[23] Marusyk, A., Janiszewska, M., Polyak, K. (2020). Intratumor heterogeneity: The rosetta stone of therapy resistance. Cancer Cell*,* 37 *(*4*),* 471–484. 10.1016/j.ccell.2020.03.007; 32289271 PMC7181408

[24] Warrier, N. M., Kelkar, N., Johnson, C. T., Govindarajan, T., Prabhu, V. et al. (2023). Understanding cancer stem cells and plasticity: Towards better therapeutics. European Journal of Cell Biology*,* 102 *(*2*),* 151321. 10.1016/j.ejcb.2023.151321; 37137199

[25] Network, C. G. A. (2015). Comprehensive genomic characterization of head and neck squamous cell carcinomas. Nature*,* 517 *(*7536*),* 576–582. 10.1038/nature14129; 25631445 PMC4311405

[26] Macosko, E. Z., Basu, A., Satija, R., Nemesh, J., Shekhar, K. et al. (2015). Highly parallel genome-wide expression profiling of individual cells using nanoliter droplets. Cell*,* 161 *(*5*),* 1202–1214. 10.1016/j.cell.2015.05.002; 26000488 PMC4481139

[27] Stahl, P. L., Salmen, F., Vickovic, S., Lundmark, A., Navarro, J. F. et al. (2016). Visualization and analysis of gene expression in tissue sections by spatial transcriptomics. Science*,* 353 *(*6294*),* 78–82. 10.1126/science.aaf2403; 27365449

[28] User Guide, CG000407 (2022). https://www.10xgenomics.com/support/spatial-gene-expression-ffpe/documentation/workflows/ffpe-v-1/steps/library-construction/visium-spatial-gene-expression-reagent-kits-for-ffpe-user-guide

[29] Picelli, S., Bjorklund, A. K., Faridani, O. R., Sagasser, S., Winberg, G. et al. (2013). Smart-seq2 for sensitive full-length transcriptome profiling in single cells. Nature Methods*,* 10 *(*11*),* 1096–1098. 10.1038/nmeth.2639; 24056875

[30] Hashimshony, T., Wagner, F., Sher, N., Yanai, I. (2012). CEL-Seq: Single-cell RNA-Seq by multiplexed linear amplification. Cell Reports*,* 2 *(*3*),* 666–673. 10.1016/j.celrep.2012.08.003; 22939981

[31] Sasagawa, Y., Nikaido, I., Hayashi, T., Danno, H., Uno, K. D. et al. (2013). Quartz-Seq: A highly reproducible and sensitive single-cell RNA sequencing method, reveals non-genetic gene-expression heterogeneity. Genome Biology*,* 14 *(*4*),* 3097. 10.1186/gb-2013-14-4-r31; 23594475 PMC4054835

[32] Jaitin, D. A., Kenigsberg, E., Keren-Shaul, H., Elefant, N., Paul, F. et al. (2014). Massively parallel single-cell RNA-seq for marker-free decomposition of tissues into cell types. Science*,* 343 *(*6172*),* 776–779. 10.1126/science.1247651; 24531970 PMC4412462

[33] Hagemann-Jensen, M., Ziegenhain, C., Chen, P., Ramskold, D., Hendriks, G. J. et al. (2020). Single-cell RNA counting at allele and isoform resolution using Smart-seq3. Nature Biotechnology*,* 38 *(*6*),* 708–714. 10.1038/s41587-020-0497-0; 32518404

[34] Ziegenhain, C., Vieth, B., Parekh, S., Reinius, B., Guillaumet-Adkins, A. et al. (2017). Comparative analysis of single-Cell RNA sequencing methods. Molecular Cell*,* 65 *(*4*),* 631–643.E4. 10.1016/j.molcel.2017.01.023; 28212749

[35] Marx, V. (2021). Method of the year: Spatially resolved transcriptomics. Nature Methods*,* 18 *(*1*),* 9–14. 10.1038/s41592-020-01033-y; 33408395

[36] Williams, C. G., Lee, H. J., Asatsuma, T., Vento-Tormo, R., Haque, A. (2022). An introduction to spatial transcriptomics for biomedical research. Genome Medicine*,* 14 *(*1*),* 68. 10.1186/s13073-022-01075-1; 35761361 PMC9238181

[37] Hernandez, S., Lazcano, R., Serrano, A., Powell, S., Kostousov, L. et al. (2022). Challenges and opportunities for immunoprofiling using a spatial high-plex technology: The NanoString GeoMx((R)) digital spatial profiler. Frontiers in Oncology*,* 12*,* 890410. 10.3389/fonc.2022.890410; 35847846 PMC9277770

[38] Hao, Y., Hao, S., Andersen-Nissen, E., Mauck, W. M. 3rd, Zheng, S. et al. (2021). Integrated analysis of multimodal single-cell data. Cell*,* 184 *(*13*),* 3573–3587.E29. 10.1016/j.cell.2021.04.048; 34062119 PMC8238499

[39] Satija, R., Farrell, J. A., Gennert, D., Schier, A. F., Regev, A. (2015). Spatial reconstruction of single-cell gene expression data. Nature Biotechnology*,* 33 *(*5*),* 495–502. 10.1038/nbt.3192; 25867923 PMC4430369

[40] Wolf, F. A., Angerer, P., Theis, F. J. (2018). SCANPY: Large-scale single-cell gene expression data analysis. Genome Biology*,* 19 *(*1*),* 15. 10.1186/s13059-017-1382-0; 29409532 PMC5802054

[41] Kannan, J., Mathews, L., Wu, Z., Young, N. S., Gao, S. (2022). CAISC: A software to integrate copy number variations and single nucleotide mutations for genetic heterogeneity profiling and subclone detection by single-cell RNA sequencing. BMC Bioinformatics*,* 23*,* 98. 10.1186/s12859-022-04625-x; 35313800 PMC8939069

[42] Serin Harmanci, A., Harmanci, A. O., Zhou, X. (2020). CaSpER identifies and visualizes CNV events by integrative analysis of single-cell or bulk RNA-sequencing data. Nature Communications*,* 11 *(*1*),* 89. 10.1038/s41467-019-13779-x; 31900397 PMC6941987

[43] Patel, A. P., Tirosh, I., Trombetta, J. J., Shalek, A. K., Gillespie, S. M. et al. (2014). Single-cell RNA-seq highlights intratumoral heterogeneity in primary glioblastoma. Science*,* 344 *(*6190*),* 1396–1401. 10.1126/science.1254257; 24925914 PMC4123637

[44] de Kanter, J. K., Lijnzaad, P., Candelli, T., Margaritis, T., Holstege, F. C. P. (2019). CHETAH: A selective, hierarchical cell type identification method for single-cell RNA sequencing. Nucleic Acids Research*,* 47 *(*16*),* e95. 10.1093/nar/gkz543; 31226206 PMC6895264

[45] Alquicira-Hernandez, J., Sathe, A., Ji, H. P., Nguyen, Q., Powell, J. E. (2019). scPred: Accurate supervised method for cell-type classification from single-cell RNA-seq data. Genome Biology*,* 20 *(*1*),* 264. 10.1186/s13059-019-1862-5 31829268 PMC6907144

[46] Boufea, K., Seth, S., Batada, N. N. (2020). scID uses discriminant analysis to identify transcriptionally equivalent cell types across single-cell RNA-seq data with batch effect. iScience*,* 23 *(*3*),* 100914. 10.1016/j.isci.2020.100914; 32151972 PMC7063229

[47] Zhang, Z., Luo, D., Zhong, X., Choi, J. H., Ma, Y. et al. (2019). SCINA: A semi-supervised subtyping algorithm of single cells and bulk samples. Genes*,* 10 *(*7*),* 531. 10.3390/genes10070531; 31336988 PMC6678337

[48] Jiao, L., Wang, G., Dai, H., Li, X., Wang, S. et al. (2023). scTransSort: Transformers for intelligent annotation of cell types by gene embeddings. Biomolecules*,* 13 *(*4*),* 611. 10.3390/biom13040611; 37189359 PMC10136153

[49] Tan, Y., Cahan, P. (2019). SingleCellNet: A computational tool to classify single cell RNA-seq data across platforms and across species. Cell Systems*,* 9 *(*2*),* 207–213.E2. 10.1016/j.cels.2019.06.004; 31377170 PMC6715530

[50] Shao, X., Yang, H., Zhuang, X., Liao, J., Yang, P. et al. (2021). scDeepSort: A pre-trained cell-type annotation method for single-cell transcriptomics using deep learning with a weighted graph neural network. Nucleic Acids Research*,* 49 *(*21*),* e122. 10.1093/nar/gkab775; 34500471 PMC8643674

[51] Dominguez Conde, C., Xu, C., Jarvis, L. B., Rainbow, D. B., Wells, S. B. et al. (2022). Cross-tissue immune cell analysis reveals tissue-specific features in humans. Science*,* 376 *(*6594*),* eabl5197. 10.1126/science.abl5197; 35549406 PMC7612735

[52] Hou, R., Denisenko, E., Forrest, A. R. R. (2019). scMatch: A single-cell gene expression profile annotation tool using reference datasets. Bioinformatics*,* 35 *(*22*),* 4688–4695. 10.1093/bioinformatics/btz292; 31028376 PMC6853649

[53] Xu, Y., Baumgart, S. J., Stegmann, C. M., Hayat, S. (2022). MACA: Marker-based automatic cell-type annotation for single-cell expression data. Bioinformatics*,* 38 *(*6*),* 1756–1760. 10.1093/bioinformatics/btab840; 34935911

[54] Bej, S., Galow, A. M., David, R., Wolfien, M., Wolkenhauer, O. (2021). Automated annotation of rare-cell types from single-cell RNA-sequencing data through synthetic oversampling. BMC Bioinformatics*,* 22 *(*1*),* 557. 10.1186/s12859-021-04469-x; 34798805 PMC8603509

[55] Grun, D., Lyubimova, A., Kester, L., Wiebrands, K., Basak, O. et al. (2015). Single-cell messenger RNA sequencing reveals rare intestinal cell types. Nature*,* 525 *(*7568*),* 251–255. 10.1038/nature14966; 26287467

[56] Jindal, A., Gupta, P., Jayadeva, Sengupta, D. (2018). Discovery of rare cells from voluminous single cell expression data. Nature Communications*,* 9 *(*1*),* 4719. 10.1038/s41467-018-07234-6; 30413715 PMC6226447

[57] Jiang, L., Chen, H., Pinello, L., Yuan, G. C. (2016). GiniClust: Detecting rare cell types from single-cell gene expression data with Gini index. Genome Biology*,* 17 *(*1*),* 144. 10.1186/s13059-016-1010-4; 27368803 PMC4930624

[58] Aran, D., Looney, A. P., Liu, L., Wu, E., Fong, V. et al. (2019). Reference-based analysis of lung single-cell sequencing reveals a transitional profibrotic macrophage. Nature Immunology*,* 20 *(*2*),* 163–172. 10.1038/s41590-018-0276-y; 30643263 PMC6340744

[59] Zhang, A. W., O’Flanagan, C., Chavez, E. A., Lim, J. L. P., Ceglia, N. et al. (2019). Probabilistic cell-type assignment of single-cell RNA-seq for tumor microenvironment profiling. Nature Methods*,* 16 *(*10*),* 1007–1015. 10.1038/s41592-019-0529-1; 31501550 PMC7485597

[60] Pliner, H. A., Shendure, J., Trapnell, C. (2019). Supervised classification enables rapid annotation of cell atlases. Nature Methods*,* 16 *(*10*),* 983–986. 10.1038/s41592-019-0535-3; 31501545 PMC6791524

[61] Wagner, D. E., Klein, A. M. (2020). Lineage tracing meets single-cell omics: Opportunities and challenges. Nature Reviews Genetics*,* 21 *(*7*),* 410–427. 10.1038/s41576-020-0223-2; 32235876 PMC7307462

[62] Trapnell, C., Cacchiarelli, D., Grimsby, J., Pokharel, P., Li, S. et al. (2014). The dynamics and regulators of cell fate decisions are revealed by pseudotemporal ordering of single cells. Nature Biotechnology*,* 32 *(*4*),* 381–386. 10.1038/nbt.2859; 24658644 PMC4122333

[63] Ji, Z., Ji, H. (2016). TSCAN: Pseudo-time reconstruction and evaluation in single-cell RNA-seq analysis. Nucleic Acids Research*,* 44 *(*13*),* e117. 10.1093/nar/gkw430; 27179027 PMC4994863

[64] Street, K., Risso, D., Fletcher, R. B., Das, D., Ngai, J. et al. (2018). Slingshot: Cell lineage and pseudotime inference for single-cell transcriptomics. BMC Genomics*,* 19 *(*1*),* 477. 10.1186/s12864-018-4772-0; 29914354 PMC6007078

[65] McCarthy, D. J., Campbell, K. R., Lun, A. T., Wills, Q. F. (2017). Scater: Pre-processing, quality control, normalization and visualization of single-cell RNA-seq data in R. Bioinformatics*,* 33 *(*8*),* 1179–1186. 10.1093/bioinformatics/btw777; 28088763 PMC5408845

[66] Matsumoto, H., Kiryu, H. (2016). SCOUP: A probabilistic model based on the Ornstein-Uhlenbeck process to analyze single-cell expression data during differentiation. BMC Bioinformatics*,* 17 *(*1*),* 232. 10.1186/s12859-016-1109-3; 27277014 PMC4898467

[67] Welch, J. D., Hartemink, A. J., Prins, J. F. (2016). SLICER: Inferring branched, nonlinear cellular trajectories from single cell RNA-seq data. Genome Biology*,* 17 *(*1*),* 106. 10.1186/s13059-016-0975-3; 27215581 PMC4877799

[68] Bendall, S. C., Davis, K. L., Amir el, A. D., Tadmor, M. D., Simonds, E. F. et al. (2014). Single-cell trajectory detection uncovers progression and regulatory coordination in human B cell development. Cell*,* 157 *(*3*),* 714–725. 10.1016/j.cell.2014.04.005; 24766814 PMC4045247

[69] Setty, M., Tadmor, M. D., Reich-Zeliger, S., Angel, O., Salame, T. M. et al. (2016). Wishbone identifies bifurcating developmental trajectories from single-cell data. Nature Biotechnology*,* 34 *(*6*),* 637–645. 10.1038/nbt.3569; 27136076 PMC4900897

[70] La Manno, G., Soldatov, R., Zeisel, A., Braun, E., Hochgerner, H. et al. (2018). RNA velocity of single cells. Nature*,* 560 *(*7719*),* 494–498. 10.1038/s41586-018-0414-6; 30089906 PMC6130801

[71] Bergen, V., Lange, M., Peidli, S., Wolf, F. A., Theis, F. J. (2020). Generalizing RNA velocity to transient cell states through dynamical modeling. Nature Biotechnology*,* 38 *(*12*),* 1408–1414. 10.1038/s41587-020-0591-3; 32747759

[72] Almet, A. A., Cang, Z., Jin, S., Nie, Q. (2021). The landscape of cell-cell communication through single-cell transcriptomics. Current Opinion in Systems Biology*,* 26*,* 12–23. 10.1016/j.coisb.2021.03.007; 33969247 PMC8104132

[73] Noel, F., Massenet-Regad, L., Carmi-Levy, I., Cappuccio, A., Grandclaudon, M. et al. (2021). Dissection of intercellular communication using the transcriptome-based framework ICELLNET. Nature Communications*,* 12 *(*1*),* 1089. 10.1038/s41467-021-21244-x; 33597528 PMC7889941

[74] Vahid, M. R., Kurlovs, A. H., Andreani, T., Auge, F., Olfati-Saber, R. et al. (2023). DiSiR: Fast and robust method to identify ligand-receptor interactions at subunit level from single-cell RNA-sequencing data. NAR Genomics and Bioinformatics*,* 5 *(*1*),* lqad030. 10.1093/nargab/lqad030; 36968431 PMC10034587

[75] Cabello-Aguilar, S., Alame, M., Kon-Sun-Tack, F., Fau, C., Lacroix, M. et al. (2020). SingleCellSignalR: Inference of intercellular networks from single-cell transcriptomics. Nucleic Acids Research*,* 48 *(*10*),* e55. 10.1093/nar/gkaa183; 32196115 PMC7261168

[76] Efremova, M., Vento-Tormo, M., Teichmann, S. A., Vento-Tormo, R. (2020). CellPhoneDB: Inferring cell-cell communication from combined expression of multi-subunit ligand-receptor complexes. Nature Protocols*,* 15 *(*4*),* 1484–1506. 10.1038/s41596-020-0292-x; 32103204

[77] Browaeys, R., Saelens, W., Saeys, Y. (2020). NicheNet: Modeling intercellular communication by linking ligands to target genes. Nature Methods*,* 17 *(*2*),* 159–162. 10.1038/s41592-019-0667-5; 31819264

[78] Jin, S., Guerrero-Juarez, C. F., Zhang, L., Chang, I., Ramos, R. et al. (2021). Inference and analysis of cell-cell communication using CellChat. Nature Communications*,* 12 *(*1*),* 1088. 10.1038/s41467-021-21246-9; 33597522 PMC7889871

[79] Zhang, Y., Liu, T., Hu, X., Wang, M., Wang, J. et al. (2021). CellCall: Integrating paired ligand-receptor and transcription factor activities for cell-cell communication. Nucleic Acids Research*,* 49 *(*15*),* 8520–8534. 10.1093/nar/gkab638; 34331449 PMC8421219

[80] Hou, R., Denisenko, E., Ong, H. T., Ramilowski, J. A., Forrest, A. R. R. (2020). Predicting cell-to-cell communication networks using NATMI. Nature Communications*,* 11 *(*1*),* 5011. 10.1038/s41467-020-18873-z; 33024107 PMC7538930

[81] Cherry, C., Maestas, D. R., Han, J., Andorko, J. I., Cahan, P. et al. (2021). Computational reconstruction of the signalling networks surrounding implanted biomaterials from single-cell transcriptomics. Nature Biomedical Engineering*,* 5 *(*10*),* 1228–1238. 10.1038/s41551-021-00770-5; 34341534 PMC9894531

[82] Puram, S. V., Tirosh, I., Parikh, A. S., Patel, A. P., Yizhak, K. et al. (2017). Single-cell transcriptomic analysis of primary and metastatic tumor ecosystems in head and neck cancer. Cell*,* 171 *(*7*),* 1611–1624.E24. 10.1016/j.cell.2017.10.044; 29198524 PMC5878932

[83] Horny, K., Sproll, C., Peiffer, L., Furtmann, F., Gerhardt, P. et al. (2023). Mesenchymal-epithelial transition in lymph node metastases of oral squamous cell carcinoma is accompanied by ZEB1 expression. Journal of Translational Medicine*,* 21 *(*1*),* 267. 10.1186/s12967-023-04102-w; 37076857 PMC10114373

[84] Song, L., Zhang, S., Yu, S., Ma, F., Wang, B. et al. (2020). Cellular heterogeneity landscape in laryngeal squamous cell carcinoma. International Journal of Cancer*,* 147 *(*10*),* 2879–2890. 10.1002/ijc.33192; 32638385

[85] Lin, C., Li, Y., Chu, Y., Lu, Y., Wei, Z. et al. (2023). Single-cell discovery of the scene and potential immunotherapeutic target in hypopharyngeal tumor environment. Cancer Gene Therapy*,* 30 *(*3*),* 462–471. 10.1038/s41417-022-00567-x; 36460803 PMC10014576

[86] Kurten, C. H. L., Kulkarni, A., Cillo, A. R., Santos, P. M., Roble, A. K. et al. (2021). Investigating immune and non-immune cell interactions in head and neck tumors by single-cell RNA sequencing. Nature Communications*,* 12 *(*1*),* 7338. 10.1038/s41467-021-27619-4; 34921143 PMC8683505

[87] Siqueira, J. M., Heguedusch, D., Rodini, C. O., Nunes, F. D., Rodrigues, M. (2023). Mechanisms involved in cancer stem cell resistance in head and neck squamous cell carcinoma. Cancer Drug Resistance*,* 6 *(*1*),* 116–137. 10.20517/cdr.2022.107; 37065869 PMC10099599

[88] Johansson, E., Ueno, H. (2021). Characterization of normal and cancer stem-like cell populations in murine lingual epithelial organoids using single-cell RNA sequencing. Scientific Reports*,* 11 *(*1*),* 22329. 10.1038/s41598-021-01783-5; 34785704 PMC8595654

[89] Xiao, Z., Dai, Z., Locasale, J. (2019). Metabolic landscape of the tumor microenvironment at single cell resolution. Nature Communications*,* 10 *(*1*),* 3763. 10.1038/s41467-019-11738-0; 31434891 PMC6704063

[90] Gavish, A., Tyler, M., Greenwald, A., Hoefflin, R., Simkin, D. et al. (2023). Hallmarks of transcriptional intratumour heterogeneity across a thousand tumours. Nature*,* 618 *(*7965*),* 598–606. 10.1038/s41586-023-06130-4; 37258682

[91] Waldman, A., Fritz, J., Lenardo, M. (2020). A guide to cancer immunotherapy: From T cell basic science to clinical practice. Nature Reviews Immunology*,* 20 *(*11*),* 651–668. 10.1038/s41577-020-0306-5; 32433532 PMC7238960

[92] Chen, J., Yang, J., Li, H., Yang, Z., Zhang, X. et al. (2021). Single-cell transcriptomics reveal the intratumoral landscape of infiltrated T-cell subpopulations in oral squamous cell carcinoma. Molecular Oncology*,* 15 *(*4*),* 866–886. 10.1002/1878-0261.12910; 33513276 PMC8024729

[93] Cillo, A., Kurten, C., Tabib, T., Qi, Z., Onkar, S. et al. (2020). Immune landscape of viral- and carcinogen-driven head and neck cancer. Immunity*,* 52 *(*1*),* 183–199.E9. 10.1016/j.immuni.2019.11.014; 31924475 PMC7201194

[94] Cheng, D., Qiu, K., Rao, Y., Mao, M., Li, L. et al. (2023). Proliferative exhausted CD8^+^ T cells exacerbate long-lasting anti-tumor effects in human papillomavirus-positive head and neck squamous cell carcinoma. eLife*,* 12*,* e82705. 10.7554/eLife.82705; 36811599 PMC9946444

[95] Chen, Y., Li, Z., Zhou, G., Sun, Y. (2021). An immune-related gene prognostic index for head and neck squamous cell carcinoma. Clinical Cancer Research*,* 27 *(*1*),* 330–341. 10.1158/1078-0432.CCR-20-2166; 33097495

[96] Woolaver, R. A., Wang, X., Krinsky, A. L., Waschke, B. C., Chen, S. M. Y. et al. (2021). Differences in TCR repertoire and T cell activation underlie the divergent outcomes of antitumor immune responses in tumor-eradicating versus tumor-progressing hosts. Journal for Immunotherapy of Cancer*,* 9 *(*1*),* e001615. 10.1136/jitc-2020-001615; 33414263 PMC7797305

[97] Ruffin, A. T., Cillo, A. R., Tabib, T., Liu, A., Onkar, S. et al. (2021). B cell signatures and tertiary lymphoid structures contribute to outcome in head and neck squamous cell carcinoma. Nature Communications*,* 12 *(*1*),* 3349. 10.1038/s41467-021-23355-x; 34099645 PMC8184766

[98] Moreno-Nieves, U., Tay, J., Saumyaa, S., Horowitz, N., Shin, J. et al. (2021). Landscape of innate lymphoid cells in human head and neck cancer reveals divergent NK cell states in the tumor microenvironment. Proceedings of the National Academy of Sciences*,* 118 *(*28*),* e2101169118. 10.1073/pnas.2101169118; 34244432 PMC8285950

[99] Puram, S. V., Mints, M., Pal, A., Qi, Z., Reeb, A. et al. (2023). Cellular states are coupled to genomic and viral heterogeneity in HPV-related oropharyngeal carcinoma. Nature Genetics*,* 55 *(*4*),* 640–650. 10.1038/s41588-023-01357-3; 37012457 PMC10191634

[100] Choi, J. H., Lee, B. S., Jang, J. Y., Lee, Y. S., Kim, H. J. et al. (2023). Single-cell transcriptome profiling of the stepwise progression of head and neck cancer. Nature Communications*,* 14 *(*1*),* 1055. 10.1038/s41467-023-36691-x; 36828832 PMC9958029

[101] Li, S., Wang, Y., Sun, R., Franceschi, D., Pan, H. et al. (2022). Single-cell transcriptome analysis reveals different immune signatures in HPV- and HPV+ driven human head and neck squamous cell carcinoma. Journal of Immunological Research*,* 2022*,* 2079389. 10.1155/2022/2079389; 36157879 PMC9507777

[102] Jiang, Y., Zhang, S., Tang, L., Li, R., Zhai, J. et al. (2022). Single-cell RNA sequencing reveals TCR^+^ macrophages in HPV-related head and neck squamous cell carcinoma. Frontiers in Immunology*,* 13*,* 1030222. 10.3389/fimmu.2022.1030222; 36389736 PMC9647120

[103] Zhang, S., Wang, B., Ma, F., Tong, F., Yan, B. et al. (2021). Characteristics of B lymphocyte infiltration in HPV^+^ head and neck squamous cell carcinoma. Cancer Science*,* 112 *(*4*),* 1402–1416. 10.1111/cas.14834; 33529452 PMC8019230

[104] Eberhardt, C. S., Kissick, H. T., Patel, M. R., Cardenas, M. A., Prokhnevska, N. et al. (2021). Functional HPV-specific PD-1^+^ stem-like CD8 T cells in head and neck cancer. Nature*,* 597 *(*7875*),* 279–284. 10.1038/s41586-021-03862-z; 34471285 PMC10201342

[105] Wei, Y., Xu, T., Li, C., Zhou, X., Qian, W. et al. (2023). CD161 characterizes an inflamed subset of cytotoxic T lymphocytes associated with prolonged survival in human papillomavirus-driven oropharyngeal cancer. Cancer Immunology Research*,* 11 *(*3*),* 306–319. 10.1158/2326-6066.CIR-22-0454; 36633583 PMC9975669

[106] Bedard, M. C., Chihanga, T., Carlile, A., Jackson, R., Brusadelli, M. G. et al. (2023). Single cell transcriptomic analysis of HPV16-infected epithelium identifies a keratinocyte subpopulation implicated in cancer. Nature Communications*,* 14 *(*1*),* 1975. 10.1038/s41467-023-37377-0; 37031202 PMC10082832

[107] Galeano Nino, J., Wu, H., LaCourse, K., Kempchinsky, A., Baryiames, A. et al. (2022). Effect of the intratumoral microbiota on spatial and cellular heterogeneity in cancer. Nature*,* 611 *(*7937*),* 810–817. 10.1038/s41586-022-05435-0; 36385528 PMC9684076

[108] Sun, L., Kang, X., Wang, C., Wang, R., Yang, G. et al. (2023). Single-cell and spatial dissection of precancerous lesions underlying the initiation process of oral squamous cell carcinoma. Cell Discovery*,* 9 *(*1*),* 28. 10.1038/s41421-023-00532-4; 36914617 PMC10011538

[109] Hu, S., Lu, H., Xie, W., Wang, D., Shan, Z. et al. (2022). TDO2^+^ myofibroblasts mediate immune suppression in malignant transformation of squamous cell carcinoma. Journal of Clinical Investigation*,* 132 *(*19*),* e157649. 10.1172/JCI157649; 35972800 PMC9525123

[110] Quah, H. S., Cao, E. Y., Suteja, L., Li, C. H., Leong, H. S. et al. (2023). Single cell analysis in head and neck cancer reveals potential immune evasion mechanisms during early metastasis. Nature Communications*,* 14 *(*1*),* 1680. 10.1038/s41467-023-37379-y; 36973261 PMC10042873

[111] Marchetti, C., de Felice, F., Romito, A., Iacobelli, V., Sassu, C. M. et al. (2021). Chemotherapy resistance in epithelial ovarian cancer: Mechanisms and emerging treatments. Seminars in Cancer Biology*,* 77*,* 144–166. 10.1016/j.semcancer.2021.08.011; 34464704

[112] Osman, A. A., Arslan, E., Bartels, M., Michikawa, C., Lindemann, A. et al. (2023). Dysregulation and epigenetic reprogramming of NRF2 signaling axis promote acquisition of cisplatin resistance and metastasis in head and neck squamous cell carcinoma. Clinical Cancer Research*,* 29 *(*7*),* 1344–1359. 10.1158/1078-0432.CCR-22-2747; 36689560 PMC10068451

[113] Zhou, L., Zeng, Z., Egloff, A. M., Zhang, F., Guo, F. et al. (2022). Checkpoint blockade-induced CD8+ T cell differentiation in head and neck cancer responders. Journal for Immunotherapy of Cancer*,* 10 *(*1*),* e004034. 10.1136/jitc-2021-004034; 35058328 PMC8772459

[114] Obradovic, A., Graves, D., Korrer, M., Wang, Y., Roy, S. et al. (2022). Immunostimulatory cancer-associated fibroblast subpopulations can predict immunotherapy response in head and neck cancer. Clinical Cancer Research*,* 28 *(*10*),* 2094–2109. 10.1158/1078-0432.CCR-21-3570; 35262677 PMC9161438

[115] Lin, M., Sade-Feldman, M., Wirth, L., Lawrence, M. S., Faden, D. L. (2022). Single-cell transcriptomic profiling for inferring tumor origin and mechanisms of therapeutic resistance. NPJ Precision Oncology*,* 6 *(*1*),* 71. 10.1038/s41698-022-00314-3; 36210388 PMC9548500

[116] Luoma, A. M., Suo, S., Wang, Y., Gunasti, L., Porter, C. B. M. et al. (2022). Tissue-resident memory and circulating T cells are early responders to pre-surgical cancer immunotherapy. Cell*,* 185 *(*16*),* 2918–2935.E29. 10.1016/j.cell.2022.06.018; 35803260 PMC9508682

[117] Sievers, C., Craveiro, M., Friedman, J., Robbins, Y., Yang, X. et al. (2023). Phenotypic plasticity and reduced tissue retention of exhausted tumor-infiltrating T cells following neoadjuvant immunotherapy in head and neck cancer. Cancer Cell*,* 41 *(*5*),* 887–902.E5. 10.1016/j.ccell.2023.03.014; 37059104 PMC10175181

[118] Zhang, Y., Liu, G., Tao, M., Ning, H., Guo, W. et al. (2023). Integrated transcriptome study of the tumor microenvironment for treatment response prediction in male predominant hypopharyngeal carcinoma. Nature Communications*,* 14 *(*1*),* 1466. 10.1038/s41467-023-37159-8; 36928331 PMC10020474

[119] Chung, C. H., Lin, C. Y., Chen, C. Y., Hsueh, C. W., Chang, Y. W. et al. (2023). Ferroptosis signature shapes the immune profiles to enhance the response to immune checkpoint inhibitors in head and neck cancer. Advanced Science*,* 10 *(*15*),* e2204514. 10.1002/advs.202204514; 37026630 PMC10214241

[120] Weber, P., Kunstner, A., Hess, J., Unger, K., Marschner, S. et al. (2022). Therapy-related transcriptional subtypes in matched primary and recurrent head and neck cancer. Clinical Cancer Research*,* 28 *(*5*),* 1038–1052. 10.1158/1078-0432.CCR-21-2244; 34965946

[121] Li, G., Jiang, Y., Li, G., Qiao, Q. (2021). Comprehensive analysis of radiosensitivity in head and neck squamous cell carcinoma. Radiotherapy and Oncology*,* 159*,* 126–135. 10.1016/j.radonc.2021.03.017; 33775714

[122] Goel, S., Bergholz, J. S., Zhao, J. J. (2022). Targeting CDK4 and CDK6 in cancer. Nature Reviews Cancer*,* 22 *(*6*),* 356–372. 10.1038/s41568-022-00456-3; 35304604 PMC9149100

[123] van Caloen, G., Machiels, J. P. (2019). Potential role of cyclin-dependent kinase 4/6 inhibitors in the treatment of squamous cell carcinoma of the head and neck. Current Opinion in Oncology*,* 31 *(*3*),* 122–130. 10.1097/CCO.0000000000000513; 30986809

[124] Chen, S. M. Y., Popolizio, V., Woolaver, R. A., Ge, H., Krinsky, A. L. et al. (2022). Differential responses to immune checkpoint inhibitor dictated by pre-existing differential immune profiles in squamous cell carcinomas caused by same initial oncogenic drivers. Journal of Experimental & Clinical Cancer Research*,* 41 *(*1*),* 123. 10.1186/s13046-022-02337-x; 35366939 PMC8976353

[125] Chen, T., Cao, C., Zhang, J., Streets, A., Li, T. et al. (2022). Histologically resolved multiomics enables precise molecular profiling of human intratumor heterogeneity. PLoS Biology*,* 20 *(*7*),* e3001699. 10.1371/journal.pbio.3001699; 35776767 PMC9282480

[126] Goltsev, Y., Samusik, N., Kennedy-Darling, J., Bhate, S., Hale, M. et al. (2018). Deep profiling of mouse splenic architecture with CODEX multiplexed imaging. Cell*,* 174 *(*4*),* 968–981.E15. 10.1016/j.cell.2018.07.010; 30078711 PMC6086938

[127] Zhang, W., Li, I., Reticker-Flynn, N. E., Good, Z., Chang, S. et al. (2022). Identification of cell types in multiplexed in situ images by combining protein expression and spatial information using CELESTA. Nature Methods*,* 19 *(*6*),* 759–769. 10.1038/s41592-022-01498-z; 35654951 PMC9728133

[128] Zahalka, A., Frenette, P. (2020). Nerves in cancer. Nature Reviews Cancer*,* 20 *(*3*),* 143–157. 10.1038/s41568-019-0237-2; 31974491 PMC7709871

[129] Zhang, Z., Liu, R., Jin, R., Fan, Y., Li, T. et al. (2019). Integrating clinical and genetic analysis of perineural invasion in head and neck squamous cell carcinoma. Frontiers in Oncology*,* 9*,* 434. 10.3389/fonc.2019.00434; 31214495 PMC6555133

[130] Schmitd, L., Perez-Pacheco, C., Bellile, E., Wu, W., Casper, K. et al. (2022). Spatial and transcriptomic analysis of perineural invasion in oral cancer. Clinical Cancer Research*,* 28 *(*16*),* 3557–3572. 10.1158/1078-0432.CCR-21-4543; 35819260 PMC9560986

